# Lipoic Acid Attenuates Lipopolysaccharide- and *Escherichia coli*-Induced Reactive Oxygen Species Production and Neutrophil Extracellular Trap Formation Without Impairing *Escherichia coli* or *Staphylococcus aureus* Killing by Human Neutrophils

**DOI:** 10.3390/ijms27136072

**Published:** 2026-07-07

**Authors:** Gisela Anay Valencia-Hernández, Mary Fafutis-Morris, Lucila A. Godínez-Méndez, Germán Muñoz-Sánchez, Marcela Guadalupe Martínez-Barajas, Andrea A. García-Contreras, Liliana Íñiguez-Gutiérrez, Vidal Delgado-Rizo

**Affiliations:** 1 Departamento de Fisiología, Centro Universitario de Ciencias de la Salud, Universidad de Guadalajara, Guadalajara 44340, Jalisco, Mexico; gisela.valencia6103@alumnos.udg.mx (G.A.V.-H.); lucila.godinez@edu.uag.mx (L.A.G.-M.); gervvan.bio@gmail.com (G.M.-S.); 2 Centro de Investigación en Inmunología y Dermatología (CIINDE), Universidad de Guadalajara, Zapopan 45190, Jalisco, Mexico; mary.fafutis@acadmicos.udg.mx; 3 Departamento Académico de Ciencias Básicas, Universidad Autónoma de Guadalajara, Zapopan 45129, Jalisco, Mexico; 4 Departamento de Microbiología y Patología, Centro Universitario de Ciencias de la Salud, Universidad de Guadalajara, Guadalajara 44340, Jalisco, Mexico; marcela.martinez@academicos.udg.mx; 5 Instituto de Nutrición Humana, Centro Universitario de Ciencias de la Salud, Universidad de Guadalajara, Guadalajara 44340, Jalisco, Mexico; andrea.garcia@academicos.udg.mx; 6 Departamento de Ciencias de la Salud, Centro Universitario de los Valles, Universidad de Guadalajara, Ameca 46600, Jalisco, Mexico; liliana.iniguez@academicos.udg.mx

**Keywords:** reactive oxygen species, alpha-lipoic acid, neutrophils, NET formation, TNF-alpha

## Abstract

Neutrophils are essential for antimicrobial defense through reactive oxygen species (ROS) production, tumor necrosis factor-alpha (TNF-α) release, and neutrophil extracellular trap formation. Lipoic acid, a redox-active antioxidant, modulates activation in human neutrophils. Neutrophils isolated from healthy donors were pretreated with lipoic acid and then exposed to lipopolysaccharide (LPS) or whole *Escherichia coli*, according to the specific assay. ROS production, NET formation, TNF-α release, bacterial killing, metabolic activity, and cell death were assessed using fluorometric assays, enzyme-linked immunosorbent assay, colony-forming unit assays, MTT reduction, and Annexin V-FITC/propidium iodide flow cytometry. Lipoic acid significantly reduced ROS production and NET formation induced by LPS and *Escherichia coli*. at 0.5 mM, lipoic acid also reduced *E. coli*-induced NET formation by approximately 50% and attenuated TNF-α release at early stimulation times. In colony-forming unit assays, lipoic acid did not significantly reduce neutrophil-mediated killing of *Escherichia coli* or *Staphylococcus aureus*. Although only neutrophil preparations with high baseline viability were used, *Escherichia coli* challenge markedly reduced cell viability during the assay; under this condition, lipoic acid pretreatment limited bacteria-induced necrosis and preserved a higher proportion of viable neutrophils. These findings indicate that lipoic acid dampens excessive oxidative and inflammatory neutrophil responses while maintaining measurable bactericidal capacity in vitro.

## 1. Introduction

Neutrophils are the most abundant leukocytes in human peripheral blood [1] and constitute one of the main effector cells of the innate immune system [2]. During early responses to bacterial and fungal infections, they function as professional phagocytes and deploy coordinated antimicrobial mechanisms, including chemotaxis, phagocytosis, degranulation, production of reactive oxygen species (ROS) [3], and neutrophil extracellular trap (NET) formation [4].

Beyond direct microbial killing, bacterial recognition and phagocytosis can also induce neutrophils to release inflammatory mediators, including tumor necrosis factor-alpha (TNF-α) and chemokines [5,6]. These mediators contribute to endothelial cell activation, increase leukocyte adhesion and transmigration, recruit additional neutrophils through chemotactic gradients, and promote monocyte/macrophage activation at the inflammatory sites [7].

Microbial recognition occurs through receptors that detect pathogen-associated molecular patterns and that bind microorganisms opsonized by complement or immunoglobulins [8]. In Gram-negative bacteria, lipopolysaccharide (LPS) is a structural component of the outer membrane and is primarily recognized by the TLR4/CD14/MD-2 complex [9]. However, stimulation with purified LPS is not equivalent to exposure to whole bacteria, since intact microorganisms such as *Escherichia coli* can simultaneously activate multiple receptors and functional responses [6].

In addition, opsonization promotes efficient interactions between neutrophils and bacteria through complement receptors, such as CR1 and CR3, which recognize particles coated with C3b or iC3b, and through Fc-gamma receptors, which recognize microorganisms opsonized with IgG [10]. During phagocytosis, assembly of the NADPH oxidase/NOX2 complex enables ROS generation, contributing to microbial elimination [8]. However, when this response is excessive or sustained, ROS can also participate in neutrophil cell-death pathways, including NET formation, apoptosis, and necrosis, depending on stimulus intensity [11].

Neutrophils have been reported to internalize approximately 47–50 bacteria per cell in vitro conditions, suggesting that a high bacterial burden can impose considerable oxidative stress on these cells [12]. An excessive or dysregulated neutrophil activity can promote inflammatory damage and contribute to endothelial and vascular injury, thrombosis, organ dysfunction, and related pathological processes [13,14]. In this context, regulating neutrophil activity without compromising its microbicidal capacity represents a relevant strategy to limit inflammatory damage. Therefore, compounds capable of modulating excessive ROS production may offer potential therapeutic benefit.

Lipoic acid, also known as alpha-lipoic acid (1,2-dithiolane-3-pentanoic acid), is found in dietary sources and endogenous mitochondrial synthesis. It is a natural organosulfur compound with antioxidant, anti-inflammatory, and redox-modulating properties [15]. Both lipoic acid and its reduced form, dihydrolipoic acid (DHLA), can participate in species scavenging, regeneration of endogenous antioxidants, and regulation of intracellular redox balance [16]. One of the main biochemical features is its ability to function as a lipoic acid/dihydrolipoic acid redox couple, which has a low standard reduction potential of approximately −0.32 V [17]. This property allows it to participate in the regulation of cellular redox balance and to contribute to the regeneration or restoration of endogenous antioxidants, including glutathione, vitamin C, and vitamin E [16].

Despite these relevant properties, evidence regarding the direct effects of lipoic acid on functionally activated neutrophils remains limited. Some studies have shown that it can modulate neutrophil differentiation, metabolism, or neutrophil-associated functions, both in vitro and in vivo [18,19,20]. In addition, other studies have reported that it reduces inflammation in tissues characterized by marked neutrophilic infiltration [21]. However, only one study has evaluated its ability to reduce reactive oxygen species production in human neutrophils stimulated with PMA, a pharmacological activator of the respiratory burst [22].

In this context, it is important to determine whether alpha lipoic acid can decrease reactive oxygen species production induced by whole bacteria, particularly under conditions in which neutrophils approach the limit of their phagocytic capacity. It is also essential to assess whether this redox modulation compromises key neutrophil functions, such as bacterial killing. This approach allows the antioxidant potential of lipoic acid to be evaluated in a more biologically relevant model, considering that neutrophils are among the major cellular sources of reactive oxygen species during the antimicrobial response.

## 2. Results

### 2.1. Lipoic Acid Does Not Significantly Reduce Neutrophil Metabolic Activity, as Assessed by the MTT Assay

**Figure 1:**
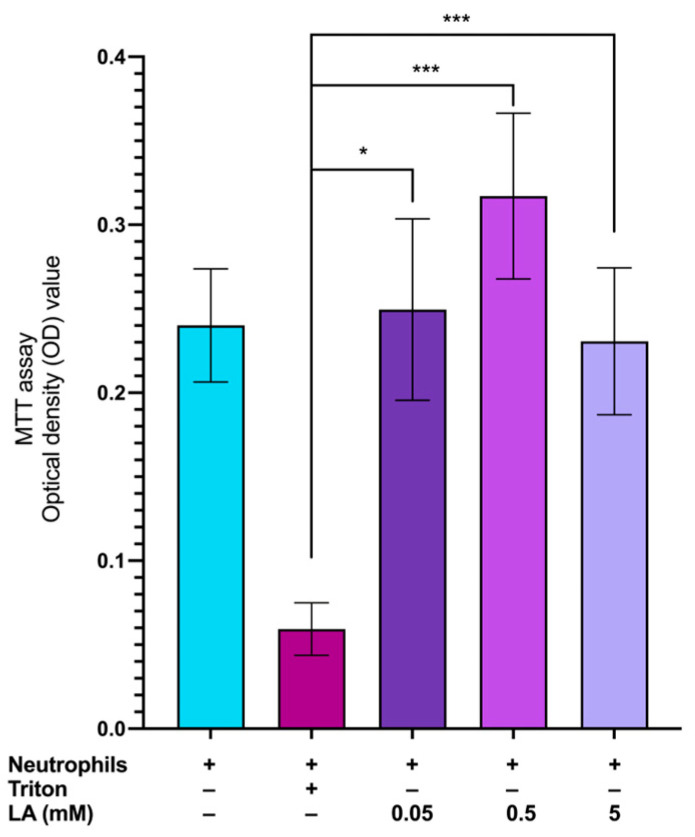
Lipoic acid (LA) does not reduce neutrophil metabolic activity at the concentrations evaluated. Lipoic acid at 0.05, 0.5, and 5 mM yielded MTT reduction levels comparable to untreated controls (left column), whereas the detergent Triton X-100 markedly reduced the MTT signal. * *p* < 0.05 and *** *p* < 0.001.

Neutrophil metabolic activity was assessed by measuring MTT reduction. Treatment with 0.05, 0.5, or 5 mM lipoic acid for 60 min did not significantly decrease MTT reduction compared with untreated neutrophils (Figure 1), indicating that lipoic acid does not impair metabolic activity. In contrast, the positive control (10% Triton X-100) caused a marked decrease in MTT reduction. These results demonstrate that lipoic acid, across the concentration range tested, does not compromise basal neutrophil metabolic activity.

### 2.2. Lipoic Acid Does Not Significantly Affect Neutrophil Viability or Induce Apoptosis/Necrosis

**Figure 2:**
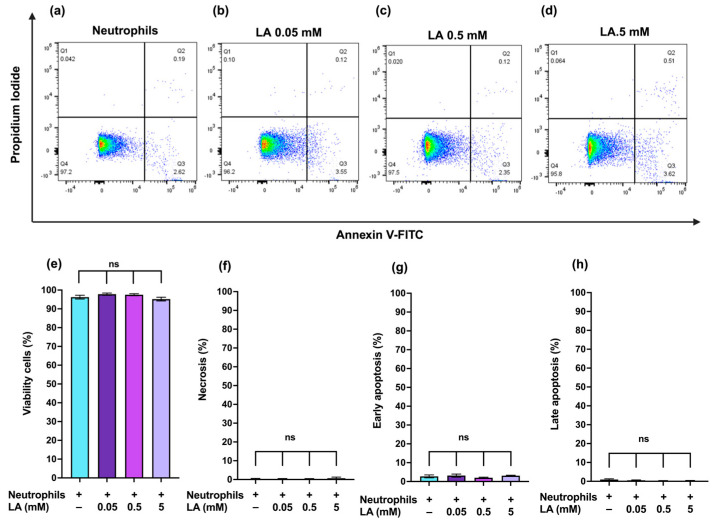
Lipoic acid does not induce apoptosis or necrosis in neutrophils at the concentrations tasted. Dot plots (**a**–**d**) and corresponding quantification (**e**–**h**) show comparable distributions of viable, early apoptotic, late apoptotic, and necrotic populations between untreated and LA-treated (0.05–5 mM) neutrophils. ns, no significant.

To determine whether lipoic acid induces cell death in neutrophils, cells were incubated with lipoic acid at 0.05, 0.5, or 5 mM for 60 min and analyzed by Annexin V-FITC/PI staining. As shown in Figure 2, lipoic acid treatment did not significantly affect the percentage of viable cells or significantly increase the percentages of necrotic, early apoptotic, or late apoptotic cells compared with untreated neutrophils. In all conditions, the viable cell population remained predominant, indicating that lipoic acid did not induce detectable apoptosis or necrosis under the experimental conditions tested.

### 2.3. Lipoic Acid Reduces NET Formation Induced by LPS and E. coli

**Figure 3:**
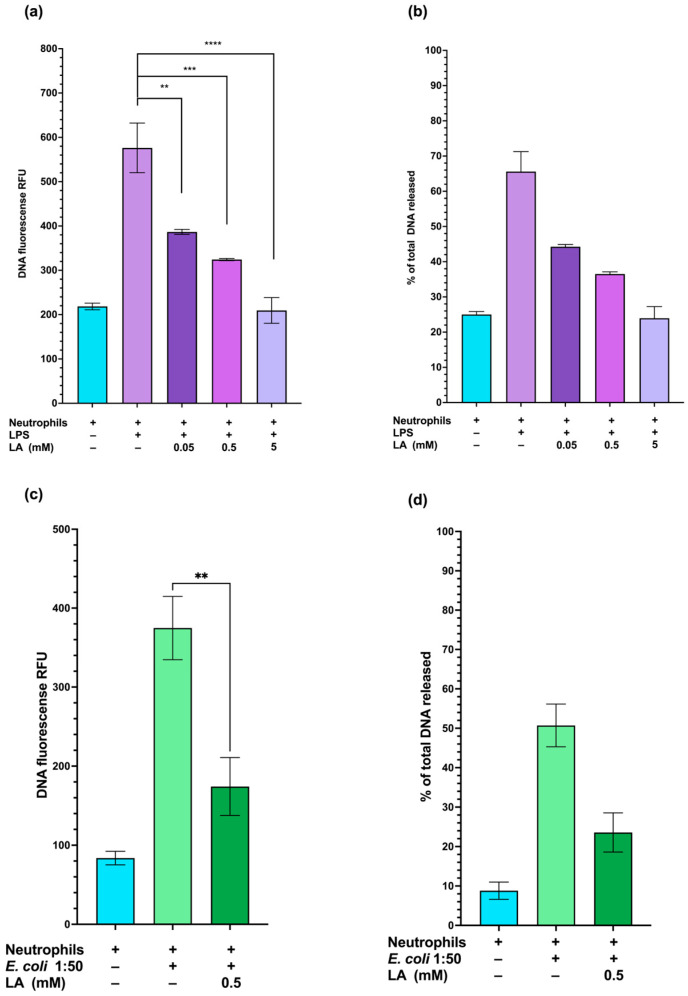
Lipoic acid reduces NET formation in neutrophils with 10 μg/mL LPS or *E. coli*. Panels (**a**,**c**) show NET-associated extracellular DNA fluorescence for LPS and *E. coli* stimulation, respectively; panels (**b**,**d**) provide the corresponding quantitative comparisons. LA treatment lowers the DNA signal relative to stimulation alone in both settings. ** *p* < 0.01, *** *p* < 0.001, and **** *p* < 0.0001.

As shown in Figure 3a,b, pretreatment with lipoic acid at 0.05 mM, 0.5 mM, and 5 mM significantly reduced LPS-induced NET formation. Based on these findings, 0.5 mM lipoic acid was selected for subsequent experiments as an intermediate concentration that showed a marked inhibitory effect on NET formation. Because LPS is a major structural component of the outer membrane of Gram-negative bacteria, we next examined whether lipoic acid also inhibits NET formation induced by whole bacteria. Neutrophils were stimulated with *E. coli* at a 1:50 neutrophil-to-bacteria ratio, and pretreatment with 0.5 mM of lipoic acid significantly reduced NET formation (Figure 3c,d).

### 2.4. Lipoic Acid Attenuates ROS Production Induced by LPS and Escherichia coli Stimulation in Human Neutrophils

**Figure 4:**
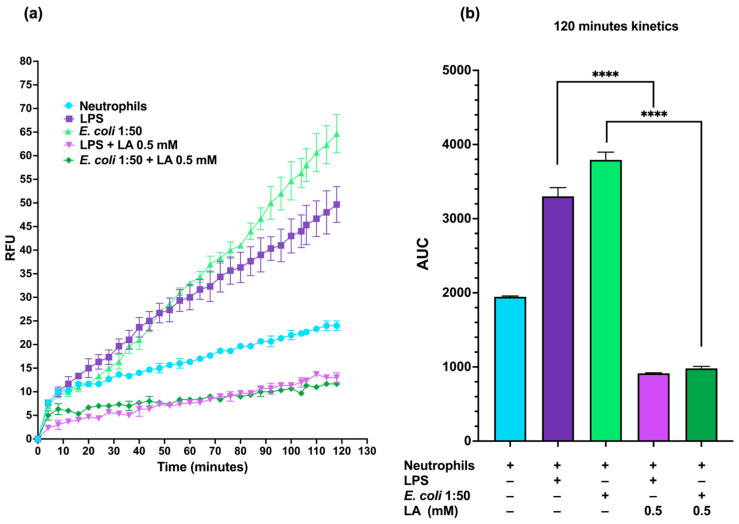
Lipoic acid attenuates ROS production in stimulated neutrophils. Real-time ROS kinetics (**a**) and the corresponding area under the curve (**b**) are shown for neutrophils stimulated with 10 μg/mL LPS or *E. coli*. Lipoic acid reduces the overall ROS response compared with stimulated controls. **** *p* < 0.0001.

In neutrophils stimulated without lipoic acid, ROS production increased markedly after 30 min of stimulation with LPS or *E. coli*. Neutrophils pretreated with lipoic acid and then stimulated with LPS or *E. coli* exhibited lower fluorescence intensity than untreated stimulated neutrophils throughout the assay (Figure 4a). Consistent with this finding, the area under the curve (AUC) over 120 min was lower in lipoic acid-pretreated neutrophils than in untreated stimulated cells (Figure 4b). These results indicate that lipoic acid attenuates ROS production induced by LPS and *E. coli* stimulation in human neutrophils.

### 2.5. Lipoic Acid Does Not Impair Neutrophil-Mediated Bacterial Killing

We performed CFU assays with *Staphylococcus aureus* and *Escherichia coli*, including bacteria-only controls for each species. Neutrophils were pretreated with lipoic acid for 1 h or left untreated and were then challenged with bacteria at a 1:50 neutrophil-to-bacteria ratio. CFU counts did not differ significantly between lipoic acid-treated and untreated neutrophils, indicating that lipoic acid does not impair bactericidal activity under the conditions tested.

### 2.6. Lipoic Acid Attenuates Early TNF-α Secretion in E. coli-Stimulated Neutrophils

**Figure 5:**
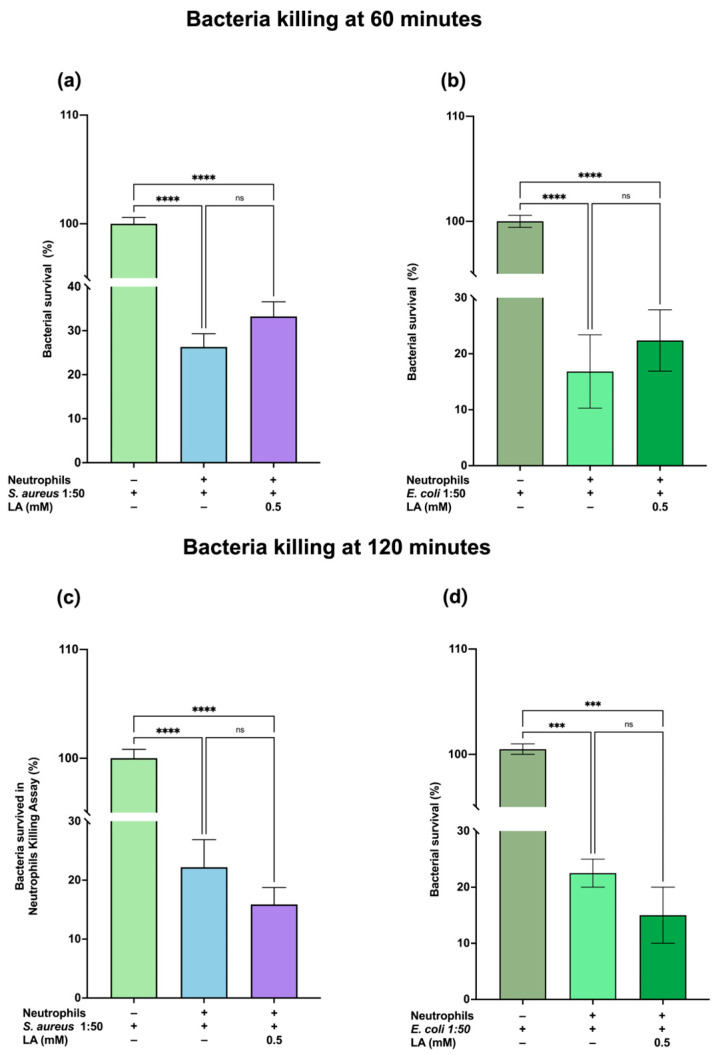
Lipoic acid does not impair neutrophil bactericidal activity. Bacterial survival (relative to bacteria-only controls) for *S. aureus* (**a**,**c**) and *E. coli* (**b**,**d**) at 60 min (**a**,**b**) and 120 min (**c**,**d**). LA-treated neutrophils maintain killing efficiency similar to that of untreated neutrophils across all conditions. *** *p* < 0.001, and **** *p* < 0.0001.

**Figure 6:**
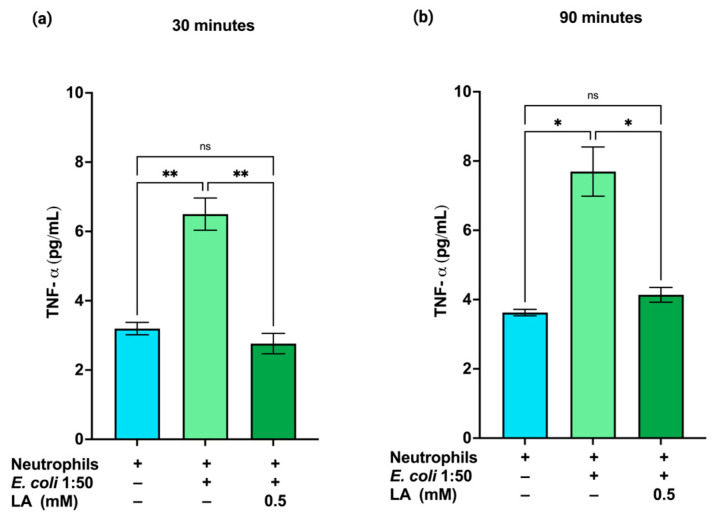
Lipoic acid reduces early TNF-α release in *E. coli*-stimulated neutrophils. TNF-α levels were measured at 30 min (**a**) and 90 min (**b**) following *E. coli* stimulation. LA-treated neutrophils show lower TNF-α levels than stimulated untreated controls. * *p* < 0.05, ** *p* < 0.01.

TNF-α is a key pro-inflammatory cytokine that promotes neutrophil activation and amplifies inflammatory responses. We therefore measured TNF-α levels in neutrophil culture supernatants 30 and 90 min after bacterial stimulation Figure 5. *E. coli* stimulation increased TNF-α release compared with untreated neutrophils. Pretreatment with lipoic acid significantly reduced TNF-α secretion relative to stimulated neutrophils alone, although levels did not fully return to basal values at 90 min. Although TNF-α levels at 90 min were slightly higher than at 30 min, the Figure 6 response remained attenuated compared with stimulated neutrophils without lipoic acid. These results indicate that lipoic acid dampens the early TNF-α response induced by *E. coli*.

### 2.7. Lipoic Acid Preserves Neutrophil Viability After Bacterial Stimulation

**Figure 7:**
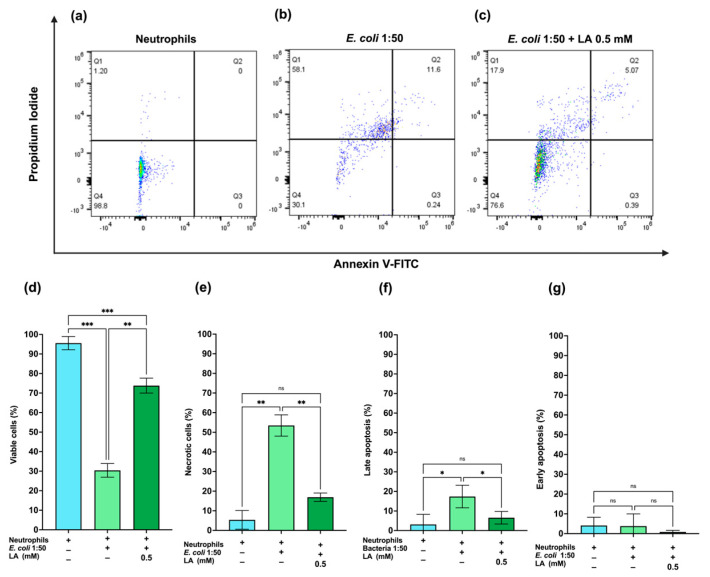
Lipoic acid preserves neutrophil viability during *E. coli* stimulation. Dot plots (**a**–**c**) and corresponding quantification (**d**–**g**) of viable, necrotic, early apoptotic, and late apoptotic populations. The addition of LA to *E. coli*-stimulated neutrophils restores the population distribution toward that of unstimulated controls. * *p* < 0.05, ** *p* < 0.01, *** *p* < 0.001.

Neutrophil viability after 2 h of bacterial stimulation was assessed by Annexin V-FITC/PI staining Figure 7. Stimulation with bacteria at a 1:50 neutrophil-to-bacteria ratio markedly reduced viability to 30%, accompanied by a predominance of necrotic cells (58%) and a minor fraction of apoptotic cells (11.6%) (Figure 7b). Under the same conditions, pretreatment with 0.5 mM lipoic acid maintained viability (76.5%), with a substantial reduction in necrotic cells (18%) and late apoptotic cells (4.92%); early apoptosis remained minimal in both conditions (Figure 7c). These results demonstrate that LA preserves neutrophil viability and reduces bacterial stimulation-induced cell death.

## 3. Discussion

The MTT assay showed that lipoic acid did not significantly affect neutrophil metabolic activity under the experimental conditions tested. Specifically, lipoic acid at 0.05 mM, 0.5 mM and 5 mM maintained MTT reduction levels comparable to those of untreated controls, suggesting that lipoic acid does not compromise basal metabolic activity.

The concentrations of lipoic acid used in this study are consistent with concentrations previously evaluated in other cell types. Huerta-Olvera et al. used HepG2 liver cells, whereas Mozaffarian et al. evaluated mitochondrial metabolic activity in isolated rat liver mitochondria [23,24].

To further confirm the safety of neutrophil exposure to lipoic acid, we performed flow cytometry with Annexin V-FITC/PI. The absence of significant changes in Annexin V- or PI-positive populations confirmed that short-term exposure to lipoic acid alone does not exert a cytotoxic effect.

Nevertheless, constitutive death, or spontaneous neutrophil apoptosis, is a central mechanism for an essential component of neutrophil homeostasis and inflammation resolution. Therefore, longer lipoic acid-only incubations could potentially influence neutrophil lifespan, possibly by reducing spontaneous apoptosis through redox-dependent mechanisms [25]. However, the biological implications of this effect would likely depend on the physiological or pathological context, such as sepsis, neutropenia, or healthy conditions. This question should be addressed in future studies specifically designed to evaluate the effect of lipoic acid on spontaneous neutrophil apoptosis over longer incubation periods.

In this study, we used both LPS, a defined Gram-negative stimulus and canonical TLR4 agonist, and whole *E. coli*, a more physiologically relevant bacterial challenge. Both stimuli induced ROS production and NET formation [4,26]. Under these conditions, lipoic acid reduced LPS-induced NET formation in a dose-dependent manner across concentrations ranging from 0.05 to 5 mM. These findings are consistent with previous reports showing that antioxidants such as vitamin C, vitamin E, glutathione, N-acetylcysteine, and resveratrol attenuate NET formation in activated neutrophils [27,28,29]. NET formation can also be induced by intact bacteria such as *E. coli*. In our model, 0.5 mM lipoic acid significantly reduced *E. coli*-induced NET formation by approximately 50%.

Alternative explanations for the decrease in extracellular DNA should also be considered. One possibility is that the lower extracellular DNA signal could reflect enhanced degradation of released NETs rather than reduced NET formation. NET degradation is mainly mediated by extracellular DNases, particularly DNase1 and DNase1-like 3, and NET clearance can also involve macrophage-dependent uptake and lysosomal degradation. However, this explanation is less likely in our experimental setting because the assays were performed with isolated neutrophils, without macrophages or other major cellular sources of NET clearance. In addition, neutrophils are not considered the principal source of the DNase1/DNase1L3-dependent extracellular degradation system involved in NET removal [30]. Therefore, although altered extracellular DNA degradation cannot be completely excluded without directly measuring DNase activity, the most plausible interpretation is that lipoic acid reduced NET formation by limiting neutrophil oxidative activation and the downstream redox-sensitive pathways required for chromatin decondensation and NET release.

Our results show that lipoic acid significantly reduced ROS levels, restoring baseline levels despite neutrophil stimulation. This finding is consistent with the observations of O’Nell et al., who demonstrated that preincubation of neutrophils with lipoic acid suppresses the respiratory burst of PMA-stimulated neutrophils without affecting glucose-6-phosphate dehydrogenase activity [22]. This suggests that its effect may occur at the level of the intracellular cascade involved in NADPH oxidase assembly or activation, rather than through a nonspecific impairment of cellular metabolism. One proposed mechanism is that reduction of lipoic acid to DHLA consumes reducing equivalents, thereby limiting NADPH availability for NOX2-dependent superoxide generation. However, in their study, they did not directly measure the NADPH/NADP+ ratio or NOX2 activity to confirm this hypothesis.

An alternative explanation is that LA/DHLA may directly influence the DCFDA-based ROS readout through its antioxidant activity. Because DCFDA does not distinguish among individual ROS and depends on the oxidation of intracellular DCFH_2_ to fluorescent DCF, LA/DHLA could reduce the fluorescence signal by scavenging ROS or by competing with DCFH_2_ for oxidizing intermediates before DCF formation. Therefore, the decrease in DCFDA fluorescence should be interpreted as a reduction in detectable oxidative activity rather than as definitive evidence of decreased ROS generation alone. Nevertheless, this possibility is unlikely to fully explain the biological effect observed. In a previous study by Muñoz-Sánchez et al. using human neutrophils, antioxidants with redox properties comparable to LA/DHLA, including GSH and NAC, also decreased DCFDA fluorescence; importantly, this reduction was accompanied by parallel decreases in independent oxidative-stress endpoints, including nitrite/nitrate levels and malondialdehyde (MDA), a marker of lipid peroxidation [28]. Thus, the concordance between reduced DCFDA fluorescence and decreased downstream oxidative damage supports the biological relevance of the ROS measurement and suggests that the effect is not merely due to a direct artifact on the DCFDA probe.

Additionally, lipoic acid reduced ROS levels in unstimulated neutrophils, as shown in Supplementary Figure S1. This suggests that lipoic acid can also modulate basal oxidative activity. This observation is relevant because neutrophils are highly reactive cells and may increase basal ROS production as a consequence of isolation, handling, and in vitro manipulation. The inclusion of vitamin C as an antioxidant reference control further supports the interpretation that this basal ROS signal is sensitive to antioxidant modulation.

One of the most noteworthy findings of this study is that lipoic acid treatment did not significantly impair the ability of neutrophils to eliminate *S. aureus* or *E. coli*, as determined by CFU assays after 60 and 120 min of co-incubation at a neutrophil-to-bacteria ratio of 1:50. The preservation of bactericidal activity at 120 min is particularly relevant because neutrophils are highly reactive and short-lived effector cells, and their functional lifespan may be further shortened under infectious or intense inflammatory conditions. Therefore, the finding that LA-treated neutrophils retained antimicrobial activity up to 2 h suggests that LA does not simply suppress neutrophil function or accelerate functional exhaustion.

This observation is also important because neutrophil-mediated bacterial clearance depends on both neutrophil concentration and bacterial burden [31]. In our study, neutrophils were challenged under high bacterial-load conditions using 6 × 10^6^ neutrophils/mL and a 1:50 neutrophil-to-bacteria ratio, representing a demanding setting in which antimicrobial capacity may become functionally saturated [31]. Under these conditions, our findings show that lipoic acid reduces ROS production and NET formation while preserving the capacity of neutrophils to continue eliminating bacteria over time.

Consistent with this modulatory profile, lipoic acid also significantly decreased TNF-α levels in culture supernatants at 30 and 90 min, suggesting attenuation of early cytokine-mediated inflammatory signaling. Thus, lipoic acid appears to dampen excessive neutrophil activation while preserving bactericidal capacity under the experimental conditions evaluated.

A plausible explanation for this preserved bactericidal activity is that lipoic acid did not induce apoptosis or necrotic loss of neutrophils during the experimental period, allowing the cells to maintain essential antimicrobial functions. This interpretation is supported by the viability assays showing no significant increase in Annexin V- or PI-positive populations after lipoic acid treatment.

An alternative, non-mutually exclusive explanation is that lipoic acid may help preserve phagocytosis-dependent bacterial clearance by limiting excessive oxidative stress and maintaining neutrophil functional integrity. However, because phagocytosis was not directly measured in the present study, this possibility should be addressed in future experiments using specific phagocytosis assays, intracellular bacterial killing assays, and compartment-specific ROS measurements. Nevertheless, future studies should determine how ROS and redox-related molecules are distributed among different cellular compartments and how this compartmentalization influences neutrophil function.

Taken together, these results demonstrate that lipoic acid attenuates key features of excessive neutrophil activation, including ROS production, NET formation, and TNF-α release, while maintaining bactericidal capacity in vitro. These findings support the concept that redox modulation can selectively dampen inflammatory responses without abolishing essential antimicrobial functions.

However, several limitations should be considered. ROS measurements were performed using DCFH-DA, which detects general oxidative activity rather than specific ROS, and extracellular DNA quantification does not exclusively distinguish NET-derived DNA from other sources of extracellular DNA. The experiments were conducted in isolated neutrophils under in vitro conditions, which do not fully reproduce the complexity of inflammatory environments involving endothelial cells, complement activation, hormonal regulation and interactions with other immune cells. Another limitation is that this study was not designed to evaluate the influence of donor sex or hormonal status on neutrophil responses. Since sex-related and hormonal factors, particularly estrogen levels, may modulate inflammatory and immune responses, future studies should include a larger donor cohort and assess hormonal profiles to determine whether lipoic-acid-mediated effects on ROS production, NET formation, TNF-α release, and bactericidal activity differ according to donor sex.

Therefore, further in vivo studies and mechanistic analyses are required to confirm these effects and define the molecular pathways involved, including NADPH oxidase activity, NF-kB signaling, MAPK pathways, and redox-dependent regulation of neutrophil activation.

## 4. Materials and Methods

### 4.1. Neutrophil Isolation

Neutrophils were obtained from a total of six healthy fasting donors, including three males and three females, aged 18–30 years. Donors had no known chronic disease, no regular medication or vitamin supplementation, and no acute infection within the preceding two weeks. All donors provided written informed consent for the exclusive use of their blood samples for experimental purposes. The study was approved by the Ethics Committee of the University Center for Health Sciences of the Universidad de Guadalajara (protocol number: 23-04).

Blood samples were processed immediately after collection. During handling, each sample was identified using a unique alphanumeric code. A double-density gradient was prepared in a 15 mL conical tube by layering blood onto Histopaque^®^ 1119 and Histopaque^®^ 1077 (Sigma-Aldrich, St. Louis, MO, USA). Samples were centrifuged at 700× *g* for 30 min at room temperature without brake to allow phase separation and recovery of polymorphonuclear cells (PMNs). PMNs were resuspended in 2 mL of PBS and used immediately for the different experiments; therefore, isolated cells were not stored. Cell viability was determined by trypan blue exclusion using a Neubauer chamber at a 1:10 dilution, and only preparations with viability greater than 95% were used.

### 4.2. Culture Conditions

Neutrophils were seeded at 2.5 × 10^5^ cells/well in 24-well plates (Costar 3526, Corning, NY, USA) and incubated in 1 mL of phenol-red-free RPMI 1640 medium (Sigma-Aldrich, R8755) supplemented with 2% sodium bicarbonate and 2% autologous serum.

### 4.3. Reagent Preparation

A fresh stock solution of lipoic acid (Sigma-Aldrich, 62320, St. Louis, MO, USA) was prepared at 24.4 mM in physiological saline containing 0.05% DMSO and protected from light. This stock solution was freshly diluted in culture medium to obtain final lipoic acid concentrations of 0.05, 0.5, and 5 mM in the wells.

Lipopolysaccharide (LPS) from *Escherichia coli* O127:B8 (Sigma-Aldrich, L3129) was reconstituted according to the manufacturer’s instructions by adding 2 mL of sterile distilled water to obtain a 5 mg/mL stock solution. The LPS stock was diluted in culture medium to obtain the final concentration used for neutrophil stimulation. LPS was used as an independent stimulus and was not replaced by whole bacteria. Whole *E. coli* and *S. aureus* were used in separate experiments, as described in the bacterial culture and bactericidal assay sections.

### 4.4. Bacterial Culture

*Escherichia coli* (ATCC 11229, strain AMC 198, Manassas, VA, USA) and *Staphylococcus aureus* (ATCC 6538, strain FDA 209, Manassas, VA, USA) were cultured in tryptic soy broth (TSB) at 37 °C for 12 h. Bacterial cells were harvested by centrifugation (1250× *g*, 10 min, room temperature), resuspended in physiological saline, and the concentration (CFU/mL) was estimated by measuring the optical density at 600 nm (OD_600_) using a previously established growth curve. Viable counts were confirmed by plating serial dilutions on standard count agar and incubating overnight at 37 °C.

### 4.5. ROS Production Assay

Intracellular ROS production was measured using the DCFDA/H_2_DCFDA Cellular ROS Assay Kit (catalog no. ab113851, Abcam, Cambridge, UK) according to the manufacturer’s protocol. Neutrophils were resuspended in PBS at 5 × 10^5^ cells/mL and pretreated with 0.5 mM lipoic acid for 60 min at 37 °C. Cells were washed three times with PBS and loaded with DCFDA for 30 min at 37 °C, washed twice with cold PBS, and resuspended in fresh phenol-red-free RPMI 1640 medium. Neutrophils were seeded into black opaque 96-well plates at 1.5 × 10^5^ cells/well in 300 µL and stimulated with 10 µg/mL LPS or *E. coli* at a neutrophil-to-bacteria ratio of 1:50. Fluorescence kinetics were monitored every 2 min for 120 min using an Synergy HTX (BioTek Instruments, Inc., Winooski, VT, USA) multimode reader at excitation 485 nm, emission 535 nm. All conditions were assayed in triplicate.

### 4.6. NET Quantification by Fluorometry

NET formation was quantified by measuring extracellular DNA fluorescence. Neutrophils were seeded at 2.5 × 10^5^ cells/well in 24-well plates (Nunclon, Delta, catalog no. 143982, Nunc A/S, Thermo Fisher Scientific Inc., Roskilde, Denmark). For LPS-induced NET formation, neutrophils were pretreated with lipoic acid at the indicated concentrations for 60 min at 37 °C and then stimulated with 10 µg/mL LPS. For whole-bacteria stimulation, neutrophils were pretreated with 0.5 mM lipoic acid for 60 min and then stimulated with *E. coli* at a neutrophil-to-bacteria ratio of 1:50. Untreated neutrophils were included as negative controls, and Triton X-100-lysed (catalog no. X100; Sigma-Aldrich Inc., St. Louis, MO, USA) neutrophils were used to determine total DNA release.

After NET induction, plates were centrifuged (500× *g*, 10 min), and 1 µL DNase I (Sigma-Aldrich) was added to each well. Plates were incubated for 30 at 37 °C and centrifuged again at 500× *g* for 10 min. Then 300 µL of each supernatant was transferred to a black 96-well plate. Next, 1 µL of SYTOX Green (5 mM stock; Thermo Fisher Scientific, S7020) was added, and the plate was incubated for 15 min at room temperature in the dark. Fluorescence was recorded with an Synergy HTX (BioTek Instruments, Inc., Winooski, VT, USA) multimode reader at excitation 485 nm, emission 527 nm, and results were expressed as relative fluorescence units (RFUs). Total DNA release was normalized to that of neutrophils lysed with Triton X-100, which was set as 100% DNA release.

### 4.7. Bactericidal Assay

Neutrophil bactericidal activity was assessed by quantifying colony-forming units (CFUs). Neutrophils 6 × 10^6^ were pretreated with 0.5 mM lipoic acid for 60 min at 37 °C and washed twice with physiological saline to remove residual compound. *E. coli* or *S. aureus* were opsonized by incubation with 10% autologous serum for 30 min at 37 °C with agitation. Neutrophils were then challenged with either bacteria at a neutrophil-to-bacteria ratio of 1:50 and incubated at 37 °C with shaking at 200 rpm for 60 or 120 min.

Following incubation, neutrophils were lysed by adding 650 µL of alkaline water, pH 11, to each 350 µL sample and incubating for 5 min at 37 °C. Suspensions were serially diluted, plated on standard count agar, and incubated overnight at 37 °C. Bacterial survival was determined by colony-forming unit counting. Bacteria cultured without neutrophils served as growth controls, and untreated neutrophils served as killing controls.

Bacterial survival (%) was calculated as the number of CFUs recovered from wells containing neutrophils and bacteria divided by the number of CFUs from the corresponding bacteria-only control, multiplied by 100. This ratio was determined separately for untreated and lipoic acid-pretreated neutrophils.

Untreated neutrophils:

$$Bacterial\;survival\;\left(\%\right)=\;\frac{CFU\;neutrophils+bacteria}{CFU\;bacteria\;only}\times 100$$

Lipoic acid -pretreated neutrophils:

$$Bacterial\;survival\;\left(\%\right)=\;\frac{CFU\;lipoic\;acid-pretreated\;neutrophils+bacteria}{CFU\;bacteria\;only}\times 100$$

### 4.8. MTT Assay

Metabolic activity was assessed using the CyQUANT™ MTT Cell Proliferation Assay Kit (I, catalog no. V13154Invitrogen, Life Technologies Corporation, Eugene, OR, USA). Neutrophils were pretreated with lipoic acid for 60 min, washed twice with PBS, and resuspended in phenol-red-free RPMI 1640 medium at 2 × 10^6^ cells/mL. Aliquots of 100 µL 2 × 10^5^ cells were seeded per well into flat-bottom 96-well plates (Thermo Fisher, 168055), and 10 µL of MTT reagent per well was added following the manufacturer’s instructions. After incubation for 3 h at 37 °C, plates were centrifuged (500× *g*, 10 min, 4 °C), supernatants were carefully removed, and formazan crystals were dissolved in 50 µL of DMSO. Absorbance was measured at 540 nm using an microplate reader (Hangzhou Allsheng Instruments Co., Ltd., Hangzhou, Zhejiang, China).

### 4.9. TNF-α Quantification by ELISA

TNF-α release was quantified in culture supernatants by enzyme-linked immunosorbent assay (ELISA). Neutrophils were pretreated with 0.5 mM lipoic acid for 60 min at 37 °C and then stimulated with *Escherichia coli* at a neutrophil-to-bacteria ratio of 1:50. Supernatants were collected after 30 and 90 min of stimulation following centrifugation to remove cells and debris. TNF-α concentrations were measured using the Quantikine HS ELISA Human TNF-α (catalog no. HSTA00E; R&D Systems, a Bio-Techne brand, Minneapolis, MN, USA) Set according to the manufacturer’s instructions. Absorbance was measured using a microplate reader, and cytokine concentrations were calculated from the standard curve and expressed as pg/mL.

### 4.10. Annexin V-FITC/PI Flow Cytometry

Neutrophil viability, apoptosis, and necrosis were assessed using the Dead Cell Apoptosis Kit with Annexin V-FITC and Propidium Iodide (catalog no. V13242, Invitrogen, Life Technologies Corporation, Eugene, OR, USA). To determine that lipoic acid alone does not induce cytotoxicity, neutrophils (2 × 10^5^ cells/mL) were pretreated with lipoic acid (0.05 mM, 0.5 mM and 5 mM) for 60 min, washed with PBS, resuspended in Annexin-binding buffer, and stained with Annexin V-FITC (5 µL) and propidium iodide (1 µL) for 15 min at room temperature in the dark.

To evaluate the effect of lipoic acid on bacteria-induced cell death, the same staining protocol was applied after 2 h of stimulation with *Escherichia coli* at a neutrophil-to-bacteria ratio of 1:50, in the presence or absence of 0.5 mM lipoic acid pretreatment. Samples were acquired using an Attune Acoustic Focusing Cytometer, with 40,000 events collected per sample, and analyzed using FlowJo™ v10.9.0.

### 4.11. Statistical Analysis

Data was analyzed using GraphPad Prism 10. Technical triplicates were averaged for each donor, and each donor was treated as an independent biological replicate. Results are expressed as mean ± SEM. Normality was assessed using the Shapiro–Wilk test. When data met parametric assumptions, statistical comparisons were performed using one-way repeated-measures ANOVA followed by Tukey’s or Dunnett’s multiple comparisons test, as appropriate. When assumptions were not met, the non-parametric Friedman test with Dunn’s post hoc test was applied. A *p*-value < 0.05 was considered statistically significant.

## 5. Conclusions

This study demonstrates that lipoic acid attenuates key inflammatory responses in human neutrophils, including ROS production, NET formation and early TNF-α secretion, while preserving neutrophil-mediated bacterial killing. Lipoic acid also reduced bacteria-induced neutrophil necrosis and improved the proportion of viable cells after bacterial stimulation. These findings support lipoic acid as a potential redox-modulating compound capable of limiting excessive neutrophil activation without abolishing essential antimicrobial functions. Further studies are required to define the molecular mechanisms involved, particularly NADPH oxidase activity, NADPH/NADP^+^ balance, and redox-sensitive inflammatory signaling pathways.

## Data Availability

The data presented in this study are available on request from the corresponding author.

## Supplementary Materials

The following supporting information can be downloaded at: https://www.mdpi.com/article/10.3390/ijms27136072/s1.

## Abbreviations

LA	Lipoic acid
DHLA	Dihydrolipoic acid
NET	Neutrophil extracellular trap
AS	Autologous serum
CFUs	Colony-forming units
DCFDA	2′7′-Dichlorofluorescin diacetate
DMSO	Dimethyl sulfoxide
ELISA	Enzyme-linked immunosorbent assay
FITC	Fluorescein isothiocyanate
LPS	Lipopolysaccharide
NF-kB	Nuclear factor kappa B
NOX2	NADPH oxidase 2
PI	Propidium iodide
RFUs	Relative fluorescence units
ROS	Reactive oxygen species
RPMI	Roswell Park Memorial Institute medium
TNF-α	Tumor necrosis factor-alpha
