# Supplementary material for: Lipoic Acid Attenuates Lipopolysaccharide- and Escherichia coli-Induced Reactive Oxygen Species Production and Neutrophil Extracellular Trap Formation Without Impairing Escherichia coli or Staphylococcus aureus Killing by Human Neutrophils
Source: Int J Mol Sci. 2026 Jul 7;27(13):6072. doi: 10.3390/ijms27136072 (PMC13362040; doi:10.3390/ijms27136072)
Supplement: Supplementary file 1 [file ijms-27-06072-s001.zip › ijms-4355846-supplementary.pdf]

Suplementary material

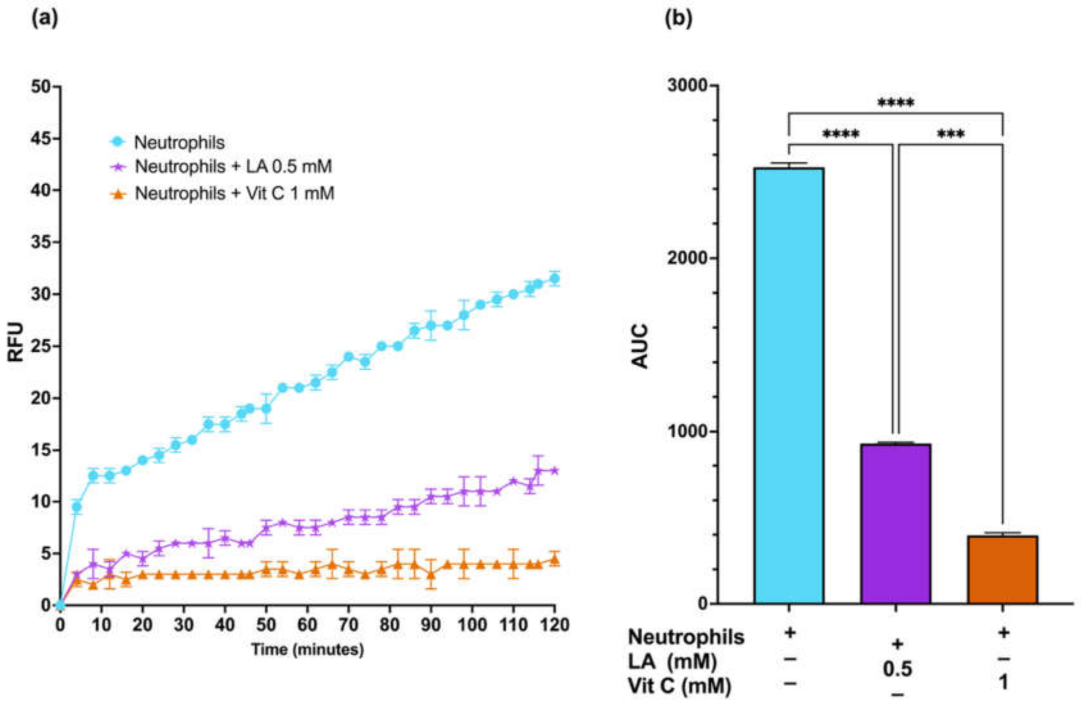

Figure S1. Effect of lipoic acid and vitamin C on basal ROS production in unstimulated human neutrophils. Real-time ROS kinetics (a) and the corresponding area under the curve (b) are shown for unstimulated neutrophils incubated with lipoic acid or vitamin C at the indicated concentrations. Both antioxidants reduced the overall basal ROS response compared with untreated unstimulated controls, suggesting modulation of basal oxidative activity under in vitro conditions. *** $p < 0.001$, and **** $p < 0.0001$.
